# Effectiveness of Electrical Muscle Elongation and Proprioceptive Neuromuscular Facilitation Programs on Muscle Flexibility and Stiffness in Young Adults with Functional Hamstring Disorder: A Randomized Clinical Trial with 4-Week Follow-Up

**DOI:** 10.3390/life15040523

**Published:** 2025-03-22

**Authors:** Carolina Jiménez-Sánchez, Rocío Fortún-Rabadán, Beatriz Carpallo-Porcar, Paula Cordova-Alegre, Luis Espejo-Antúnez, María Ortiz-Lucas

**Affiliations:** 1 Department of Physiotherapy, Faculty of Health Sciences, Universidad San Jorge, 50830 Villanueva de Gállego, Zaragoza, Spain; cjimenez@usj.es (C.J.-S.); bcarpallo@usj.es (B.C.-P.); pcordova@usj.es (P.C.-A.); 2 Aragón Health Research Institute, 50009 Zaragoza, Zaragoza, Spain; 3 Department of Medical-Surgical Therapy, Faculty of Medicine and Health Sciences, University of Extremadura, 06006 Badajoz, Badajoz, Spain; luisea@unex.es; 4 Faculty of Education, Universidad Europea de Madrid, 28670 Madrid, Madrid, Spain; m.ortiz.lucas@facultyue.es

**Keywords:** muscle disorder, hamstring, electrical stimulation, stretching, flexibility, stiffness

## Abstract

Background: Adequate hamstring flexibility is crucial for musculoskeletal health as increased muscle tone can lead to stretch-type injuries, muscle weakness, dysfunctional neuromuscular control, postural changes, and lower back pain. The aim was to compare the effectiveness of a program based on Electrical Muscle Elongation (EME), Proprioceptive Neuromuscular Facilitation (PNF), and no intervention in improving flexibility and viscoelastic properties of hamstring and quadriceps muscles in active young adults with functional hamstring disorder (type 2B according to the Munich Consensus). Methods: Sixty-five participants (45 male, 20 female) were randomly assigned to three groups: the EME group (*n* = 21) received a simultaneous combination of interferential current and stretching, the PNF group (*n* = 22) underwent active stretching, and the Control group (*n* = 22) received no intervention. Hamstring and quadricep flexibility and muscle stiffness were measured in both limbs at baseline, post-intervention, and at the 4-week follow-up. Results: The EME group showed significant improvements in hamstring flexibility in the left limb compared to the Control group and in some myotonometric variables of the quadriceps muscle compared to the PNF and Control groups (*p* < 0.05). Within-groups differences indicated higher improvements in the EME group. Conclusions: This study suggests that EME may offer greater benefits than PNF stretching in young adults with functional hamstring disorder.

## 1. Introduction

Hamstring muscle injuries are common among people who play sports, especially those involving kicking or a rapid change in running speed [1,2]. According to the latest evidence, they represent up to 25% of all muscle injuries [2], with the myotendinous unit being the site accounting for most of the hamstring injuries [1,3] and rehabilitation needs [4]. Moreover, re-injuries typically occur in this muscle group, requiring a longer period to return to competition [5].

The multifactorial nature of this clinical entity is highlighted. The mechanisms of stretch-type strain injuries at the hamstrings include reaching an extensive hip flexion with an extended knee, exceeding the limits of the muscle-tendon unit [2]. Among others, several anatomical and neuromuscular aspects such as proprioceptive/neuromuscular deficits, strength imbalances or stiffness are considered risk factors [6,7]. However, reduced flexibility and increased muscle tone along the length of the muscle belly are common findings among individuals who suffer from functional hamstring disorders [8], which also underly most strain injuries [9]. Overall, these facts emphasize the need for optimal hamstring extensibility during eccentric contraction to avoid injuries.

Adequate hamstring flexibility is essential for the correct functioning of the musculoskeletal system, given their clinical relevance in postural control mechanisms [10,11]. Indeed, functional hamstring disorders have been associated with a reduction in muscle strength and impaired quadricep activation, as well as postural changes leading to low back hyperlordosis and pain [12,13]. Therefore, it seems crucial that efforts are directed towards the design of preventive and management strategies according to the type of hamstring injury [14,15].

Previous studies have suggested that the inclusion of stretching exercises in prevention and rehabilitation programs is beneficial to ensure or restore adequate hamstring function [8,15,16]. During the last decade, different stretching modalities such as static [17] and dynamic [18] stretching, neurodynamic sliding technique [19], Electrical Muscle Elongation (EME) [20], or Proprioceptive Neuromuscular Facilitation (PNF) [21] have been proposed as strategies for immediate increases in hamstring length. However, evidence is very limited regarding the changes induced by stretching techniques in muscle viscoelastic properties such as stiffness [22]. Furthermore, studies investigating their effects in a follow-up period after interventions are lacking, and these seem to be relevant needs given the role of altered neuromuscular control mechanisms in functional muscle disorders [8].

In recent years, EME technique has been postulated as a novel physiotherapeutic intervention that has shown promising results in improving the pain, range of movement, and pressure pain threshold in adults with functional hamstring disorders [23]. This electrotherapeutic procedure consists of combining a passive stretching technique with a simultaneous electrical current that stimulates a slight isometric muscle contraction and contraction of the antagonist muscle group [24]. Therefore, the refractory period of the muscle fiber is delayed, allowing the temperature of the collagen matrix to rise. This may induce a greater ulterior muscle relaxation and better gliding capacity, achieving more flexibility of the deeper layers of the muscle connective tissue [25]. By these effects, this innovative way of application could also improve muscle stiffness [22]. However, this technique has not been sufficiently analyzed, and its effects, both in the target hamstring muscles presenting functional disorders and in the antagonist muscles, must be investigated.

Therefore, this study aimed to assess the effectiveness of a stretching program based on EME compared to PNF stretching and no intervention on muscle extensibility and viscoelastic properties of hamstring and quadriceps muscles in active young adults with functional hamstring disorder.

## 2. Materials and Methods

### 2.1. Study Design

The study was a randomized, single-blinded controlled clinical trial conducted in accordance with the Declaration of Helsinki. This study was approved by the Ethics Committee of Aragon (N°PI16/0033) and registered at ClinicalTrials.gov Protocol Registration System (reference: NCT03084341) following the CONSORT (Consolidated Standards of Reporting Trials) guidelines [26].

### 2.2. Participants

Young adult volunteers from the local community with limited hamstring muscle extensibility were recruited to participate in this study. All participants provided signed informed consent prior to the start of the procedure.

The inclusion criteria were as follows: (1) physically active participants over 18 years of age (2) with hamstring disorder classified as 2b according to the consensus of Munich [8] (diagnosed by a doctor) at this time and (3) presenting less than 60° of knee extension in the Active Knee Extension (AKE) test, as well as a Straight Leg Raise (SLR) test of ≤80°. The exclusion criteria were as follows: (1) participation in an organized hamstring stretching program, (2) pain or musculoskeletal injuries or recent surgery in the abdominal or lumbar spine and/or lower limbs, and (3) no evidence of neurological disorder.

The sample size was previously calculated to detect a difference of 10.5° with a Standard Deviation of 10.1° on the AKE test. The minimal number of subjects required to achieve a power of 0.8 and an alpha level of 0.2 was calculated to be 19 in each group including, 20% more to cover possible losses.

### 2.3. Randomization and Blinding

A researcher who was not involved in recruitment used a website (www.randomizer.org) (accessed on 17 March 2017) to randomly assign participants (using block randomization, 1:2) to one of the following groups: Electrical Muscle Elongation (EME) group, Proprioceptive Neuromuscular Facilitation (PNF) group, and Control (CT) group. Randomization was performed in numbered, sealed and opaque envelopes.

The study was conducted by two physiotherapists with more than 15 years of experience: One blinded researcher recorded the measurements at baseline, after the intervention and at the end of the follow-up period, while another researcher performed the interventions.

The allocation process was conducted in a protected area to ensure that both the examiner and the intervention provider remained blind.

### 2.4. Procedure

At the beginning of the study, participants completed a socio-demographic questionnaire. All outcomes were measured at baseline (T0), after the last intervention (T1), and 4 weeks after the last intervention (T2).

Before each session, hamstring flexibility was assessed according to the criteria previously described (<60° AKE) [27]. In case there was shortening, the corresponding intervention was performed until the values considered as normal (≥60° in the Active Knee Extension (AKE) test and ≥80° in the Straight Leg Raise (SLR) test) were reached, with a maximum of 8 sessions (2 per week). Otherwise, if no shortening occurred, the intervention was finished at that point.

Each testing session lasted between 45 and 60 min and was conducted at approximately the same time slot for each participant. The interventions were implemented in accordance with the recommendations of the Template for Intervention Description and Replication (TIDieR) checklist [28].

### 2.5. Outcome Measures

All measurements were carried out by the same trained physiotherapist. At the beginning of the first session, demographic and clinical data including age, sex, height, weight, body mass index (BMI), and level of regular physical activity using the International Physical Activity Questionnaire (IPAQ, short version) were recorded. The participants did not perform sport, warm-up or stretching exercises before the assessment and were blinded to all measurements. All verbal instructions and explanations were standardized.

#### 2.5.1. Hamstring Flexibility Assessment

For the Active Knee Extension (AKE) test, the participants were assessed in a supine position on a table, facing a rectangular wooden frame attached to the table. The thigh of the tested limb was in contact with the wooden frame, with the hip and knee joints flexed at 90° and the ankle joint in a neutral position. The contralateral lower limb was secured extended and in neutral rotation to the table using a strap across the thigh. A standard universal goniometer (Baseline^©^, Fabrication Enterprises, Inc., Elmsford, NY, USA) was placed over the lateral condyle of the femur with the proximal arm aligned along the thigh in the direction to the greater trochanter and the distal arm aligned over the leg in the direction to the lateral malleolus. After being positioned, participants were asked to extend the knee until they felt a strong resistance and to hold this final position for 2 to 3 s to allow the goniometric reading. The recorded result corresponded to the amplitude of the knee maximum extension in degrees, starting from the initial test position (knee flexed at 90°, which corresponded to the goniometric value 0°) [29,30,31].

The AKE test has an intraclass correlation coefficient (ICC) of 0.87–0.94, a standard error of measurement (SEM) of 2.6–2.9°, and a minimal detectable difference (MDD) of 7–8° [31].

The Straight Leg Raise (SLR) test was carried out with the participants lying supine on a table. The contralateral limb was secured with a strap over the thigh to maintain it extended and in neutral rotation. A standard universal goniometer was placed over the greater trochanter of the tested limb, and the goniometer arms were aligned along the midline of the pelvis and with the lateral femoral condyle. Then, the participants raised the tested lower limb with the knees fully extended and the foot in a neutral position slowly to the point that felt a strong resistance in hamstring muscles or when pelvic rotation was observed. When the participant reached the maximum hip flexion with the knee extended, the angle of the hip joint was measured. As described for the AKE test, the participants also held the final position of the SLR test for 2 to 3 s to allow the goniometric reading [20].

The SLR test has an intraclass correlation coefficient (ICC) of 0.93–0.97, a standard error of measurement (SEM) of 2.2–2.6°, and a minimal detectable difference (MDD) of 6–7° [31].

The difference in the flexibility of the right/left hamstrings was calculated to measure the asymmetry in the length of the hamstrings. Differences of more than 10–15% between limbs are considered an injury risk factor [32].

#### 2.5.2. Quadricep Flexibility Assessment

Quadricep flexibility was assessed in a prone position on a table. A standard universal goniometer (Baseline©, Fabrication Enterprises, Inc., Elmsford, NY, USA) was placed over the lateral condyle of the femur with the proximal arm aligned along the thigh in the direction to the greater trochanter and the distal arm aligned over the leg in the direction to the lateral malleolus. The assessor slowly bent the participant’s knee so that the heel approached the buttock. Attention was taken to ensure that there was no movement of the lumbar spine or pelvis or cramping of the hamstrings and that the thighs remained parallel. The subject was asked to report as soon as the first stretch sensation was experienced in the quadriceps muscle. The recorded result corresponded to the amplitude of the maximum flexion of the knee in degrees (°), starting from the initial test position (0°). Moreover, in this final position, the closest distance from the relaxed buttock to the heel with the ankle passively plantar flexed was measured with a tape measure (centimetres) [31,33].

#### 2.5.3. Muscle Stiffness Assessment

Myotonometric parameters were assessed using the MyotonPRO device (Müomeetria AS, Tallinn, Estonia). The device provides a controlled preload of 0.18 N for pre-compression of the tissues and then exerts an additional 15 ms impulse of 0.40 N of mechanical force, which induces a damped natural oscillation of the tissues. Recorded parameters by the testing probe were as follows: (1) oscillation frequency (Hz) as an indicator of muscle tone, which characterizes the resting level of tension in the tissue; (2) logarithmic decrement (arbitrary unit), which is inversely proportional to elasticity, is considered to be the ability of the muscle to restore its initial shape after being deformed; and (3) stiffness (N/m), which reflects the resistance of the tissue to the force that changes its shape [34].

Each testing site on the muscle belly was located using a tape measure and marked using a permanent dermographic pencil. In the supine position, the rectus femoris (RF) site was located and marked at two-thirds of the way between the anterior superior iliac spine and the superior pole of the patella. In the prone position, the biceps femoris (BF) site was located and marked midway between the ischial tuberosity and the head of the fibula [35].

A measurement of 10 consecutive single impulses (multiscan mode) with an interval time of 1 s was completed in each site. The mean data of each series were accepted if the coefficient of variation of the measurement set was inferior to 3% [34].

The MyotonPRO has an intraclass correlation coefficient (ICC) of 0.99 for RF and BF [36].

### 2.6. Interventions

#### 2.6.1. Proprioceptive Neuromuscular Facilitation Technique

**Figure 1:**
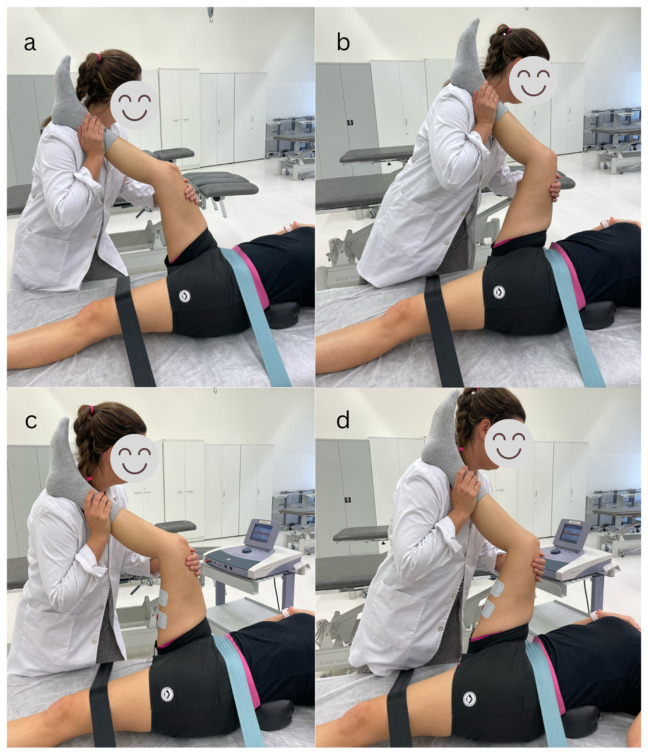
PNF (**a**,**b**) and EME technique progression (**c**,**d**).

The PNF group carried out the PNF stretching technique. Participants were supine on a table and secured with straps over the contralateral lower limb, which was extended and in neutral rotation, and over the anterior superior iliac spine. A lumbar roll was placed under the participants lower back during the stretching intervention to maintain anterior pelvic tilt during the procedure [21]. The stretching movement was performed passively by maintaining this knee position with the ankle in a relaxed position and increasing the hip flexion until a feeling of resistance appeared. After that, the participant carried out an isometric contraction of the agonist muscle (hamstrings) against resistance for 3 s, followed by a concentric contraction of the antagonist muscle (quadriceps) for 3 s. Hereafter, hip flexion was passively increased again until the participant felt a new stretching sensation. The PNF procedure was repeated four times [37] (Figure 1).

#### 2.6.2. Electrical Muscle Elongation Technique

The participants assigned to the EME group received a bipolar interferential current application with a frequency of 4 kHz and a frequency modulation amplitude of 100 Hz using an electrotherapy equipment (Sonopuls 692^®^, Enraf-Nonius, Delft, The Netherlands) and following the procedure described by Espejo (2022) [25]. Two self-adhesive electrodes (7.5 cm × 10 cm and 75 cm^2^ surface area; type Pals Platinum©, Axelgaard Manufacturing Co. Ltd., Fallbrook, CA, USA) were placed longitudinally along the hamstring muscles and covered the biceps femoris and semitendinosus muscles. Participants were placed supine on a table and secured with straps over the contralateral lower limb, which was extended and in neutral rotation, and over the anterior superior iliac spine. During the stretching intervention, a lumbar roll was placed under the participant’s lower back to maintain anterior pelvic tilt during the procedure [21]. The stretched limb was placed over the physiotherapist’s shoulder with the hip joint flexed and the knee slightly bent to avoid nerve strain. The stretching movement was performed passively by maintaining the knee position with the ankle in a relaxed position and increasing the hip flexion until a sensation of resistance was felt. At this point the intensity of the electrical current was increased until a tolerable hamstring contraction was produced. Then, the participant carried out a concentric contraction of the antagonist muscle (quadriceps) for 3 s. Hereafter, hip flexion was passively gained, reaching a new stretching sensation. At this point, the intensity of the current was increased again until the stretching sensation disappeared. This cycle was repeated four times [20,23,37] (Figure 1).

#### 2.6.3. No Intervention

Participants randomized to the CT group received no intervention.

### 2.7. Statistical Analysis

The data were analyzed using the Statistical Package for the Social Sciences (SPSS) V.28 (SPSS Inc., Chicago, IL, USA). The normal distribution of the quantitative variables was tested using the Kolmogorov–Smirnov test. Descriptive statistics were expressed as mean ± SD or median [interquartile range] for continuous parameters and frequency (%) for categorical data. Baseline measurements were compared between groups using one-way ANOVA or the Kruskal–Wallis test and the chi-square test.

Between- and within-intervention analyses were conducted using one-way ANOVA and mixed-design ANOVA with Bonferroni post hoc pairwise comparisons when a normal distribution was found. Assuming a non-normal distribution, non-parametric analyses were performed using the Kruskal–Wallis test and the Mann–Whitney U test for between-intervention comparisons and the Friedman test with the Tukey post hoc test to highlight within-intervention differences. In the Mann–Whitney U test, type I error will be divided by the number of tests performed. The significance level was established at *p* < 0.05.

Furthermore, the effect size was calculated through Cohen’s d coefficient and interpreted as small (d = 0.2), medium (d = 0.5), or large (d > 0.8) [38].

An intention-to-treat (ITT) procedure was carried out.

## 3. Results

**Figure 2:**
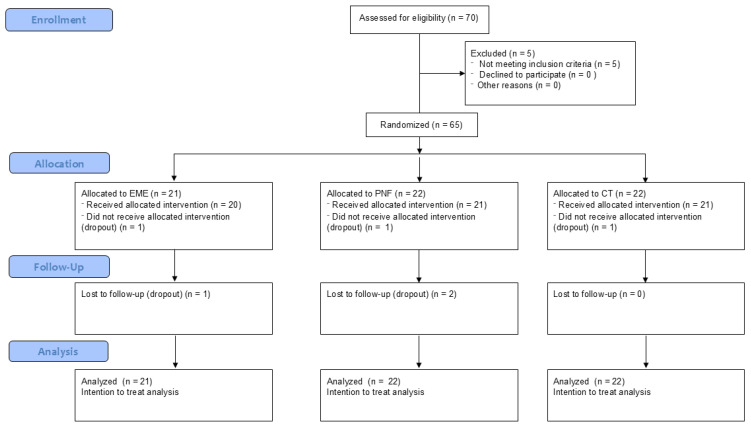
Flow chart of the study.

A total of 65 participants (45 males and 20 females; age 23.0 ± 4.4 years; stature 1.76 ± 0.1 m; weight 71.0 ± 13.0 kg; body mass index 22.8 ± 2.8 kg/m^2^) were included in this study (Table 1). The study flow chart can be seen in Figure 2.

In the initial assessment, there were significant differences between groups in terms of dominance, as well as between the right and left limbs in the values of the AKE (right 49.92° ± 8.95; left 48.08° ± 8.37) and SLR (right 62.38° ± 8.15; left 60.54° ± 8.20) tests for all participants (*n* = 65) (*p* < 0.05). However, these differences were less than 4%, and no association was found with the dominance of the participants (*p* > 0.05).

The percentage of participants reporting a high-intensity physical activity level was lower in the EME group compared with PNF and CT groups (*p* < 0.05) (Table 1). The characteristics of the sample are shown in Table 1.

The number of intervention sessions for each group was comparable for the EME and PNF groups, receiving 6.65 ± 1.75 and 6.45 ± 1.76 sessions, respectively. There were no statistically significant differences between the two experimental groups (*p* > 0.05).

### 3.1. Hamstring Flexibility

**Figure 3:**
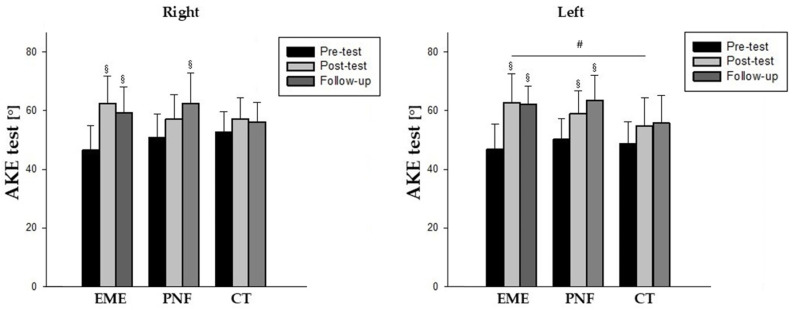
Comparative change in AKE test [°]. Abbreviations: EME, Electrical Muscle Stretching Group; PNF, Proprioceptive Neuromuscular Facilitation Group; CT, Control Group; ^§^ significance using Friedman test; ^#^ significance using Mann–Whitney U test.

In the between-groups analysis, the comparison of pre- and post-intervention values showed a significant increase in the EME group as compared to controls for the AKE test of the left limb (*p* = 0.016), with a large effect size (d = 0.809) (Table 2) (Figure 3). No other significant difference was found between groups, either for the SLR test values (*p* > 0.05).

In the within-groups analysis, the EME group showed a significant increase in the AKE test of 16° for both sides after the intervention, with a large effect size, which was maintained at follow-up (*p* < 0.001). In the PNF group, the AKE test improved only on the left side after the intervention (*p* = 0.003; d = 1.083), while at follow-up significant increases were found on both sides compared to baseline (*p* < 0.001) with large effect sizes (Table 2) (Figure 3).

Regarding the SLR test, EME and PNF groups showed improvements in both sides after the intervention, with large effect sizes, which persisted at follow-up (*p* < 0.01) (Table 2).

### 3.2. Quadricep Flexibility

No significant changes were found between the groups in the flexibility of the quadriceps in relation to the maximum flexion of the knee and the distance between the buttock and heel (*p* > 0.05) (Table 3).

Regarding within-groups analysis, a significant improvement (*p* = 0.045) in left knee flexion was found in the EME group with a moderate effect size. Furthermore, statistically significant reductions (*p* < 0.001) were found for the buttock–heel distance for the EME group after the intervention and follow-up with moderate–large effect sizes, whereas the PNF group only exhibited significant improvements (*p* = 0.043; d = 0.666) at follow-up compared to baseline (Table 3).

### 3.3. Hamstring Muscle Stiffness

After the intervention, there were no significant differences between the groups in oscillation frequency, decrement, and stiffness variables (*p* > 0.05) (Table 4). However, there were large effect sizes after the intervention in the stiffness variable in the EME group compared to the CT group (right: d = 0.811; left: d = 1.169) and in the left bicep femoral muscle at follow-up between the EME group and the PNF group (d = 0.859) with higher stiffness in both assessments in the EME group.

The within-groups analysis revealed significant differences in frequency and stiffness with higher values for the EME group after the intervention on both sides (*p* < 0.05) with moderate effect sizes, but these changes were not significant at follow-up. The PNF and Control groups did not change significantly over time (*p* > 0.05) (Table 4).

### 3.4. Quadriceps Muscle Stiffness

The between-groups analysis revealed significant differences with large effect sizes for the decrement variable with higher values after the intervention between the EME group compared to the PNF group (*p* = 0.038; d = 0.800) and the CT group (*p* = 0.005; d = 0.923) (Table 5). In addition, significant differences with moderate effect sizes in the stiffness variable were observed in both limbs after the intervention between the EME group and the PNF group (right: *p* = 0.010; d = 0.563; left: *p* = 0.024; d = 0.723) and the EME group and the CT group (right: *p* = 0.008; d = 0.742; left: *p* = 0.049; d = 0.636). At follow-up, statistically significant differences were only found for the right quadriceps muscle (*p* = 0.009; d = 0.638) (Table 5).

In the within-groups analysis, there were no significant differences in the myotonometric variables for any inter-group comparisons over time (*p* > 0.05) (Table 5).

## 4. Discussion

The aim of this study was to evaluate the effects of an 8-week program based on EME versus PNF techniques, and no intervention, on the viscoelastic properties of the hamstring and quadricep muscles in young adults with functional hamstring disorder. Additionally, the maintenance of these effects was studied after a 4-week follow-up period.

Stretching techniques constitute the most recommended intervention to improve hamstring neuromuscular muscle disorders by means of increasing flexibility [15]. However, to our knowledge, this is the first study to analyze the effects of different stretching modalities on a variety of relevant muscle properties, also measuring the impact on antagonist muscles. Furthermore, a non-intervention Control group and a follow-up assessment were accomplished.

The between-groups comparison of hamstring extensibility showed a significant improvement in AKE for the EME group compared with the non-intervention controls, which was not achieved by the PNF group. Nevertheless, this difference was only significant for the left limb. The minimum asymmetries evidenced at baseline (pre-test measures) could have influenced the results. Alternatively, as observed in other work [39], the dominance of the participants may be a more plausible explanation since most participants were right-dominant and the left side showed the most significant improvements in flexibility. Accounting for such a relevant finding, further studies on EME are warranted to address this hypothesis by including a balanced proportion of left-dominant subjects. Moreover, this consideration may be generalized to other investigations aimed at increasing muscle flexibility, irrespective of the selected modality. On the other hand, the increased hamstring flexibility achieved in the EME group in contrast with non-intervention controls after the intervention was not significantly maintained in the follow-up. It was previously demonstrated that physical activity levels can influence muscle viscoelastic properties, with more physically active individuals presenting higher levels of flexibility and lower levels of muscle stiffness [40,41]. In the present study, the EME group mostly performed moderate-intensity physical activity, while the PNF and Control groups performed significantly higher-intensity (vigorous) physical activity. Then, it is reasonable to hypothesize that the increases in flexibility observed in the PNF and non-intervention groups could have been enhanced for this reason, partially diluting the relative achievements of the EME group. However, the methods here conducted do not specifically allow this contrast since physical activity was not assessed as continuous outcome. Other authors comparing EME and PNF techniques found no influence of physical activity in hamstring flexibility, although only 30 subjects were analyzed, and hamstring disorders were not included [42]. In view of this, our results suggest the need for further research to elucidate whether physical activity can impact the effectiveness of stretching programs conducted to restore muscle flexibility in functional hamstring disorders.

The within-groups results demonstrated that both EME and PNF can increase the flexibility of shortened hamstring muscles requiring a similar number of sessions, although some aspects need to be considered. First, the EME program produced a significant and bilateral increase in hamstring flexibility as measured by the AKE and SLR tests. The efficacy of EME by an interferential current stimulation has been previously contrasted, whereby attributed to a decrease in the orthosympathetic activity and an increase in temperature in the collagen matrix [23,43]. Both underlying effects were pointed out to promote slippage of the muscle connective tissue, thus increasing the hamstring flexibility when combined with a stretching technique. The findings shown here support and reinforce this evidence by also reporting an increase in the hip range of motion through the SLR test. Moreover, these significant benefits remained during the follow-up period up to 4 weeks after the intervention, which is a novel finding for the EME applied through interferential current. Another study [44] also reported increases in hamstring muscle flexibility after follow-up by means of EME while applying transcutaneous electrical nerve stimulation (TENS). Low-frequency (TENS) and medium-frequency (interferential) currents have the same main effects at the neuromuscular level, such as the ability to induce neuromuscular relaxation, as well as activating sensory fibers leading to higher pain thresholds [45,46]. Through these mechanisms, EME could help achieve greater ranges of motion during stretching, which added to the capacity of the electrical current to modulate viscoelastic properties and would explain the effects after the intervention, as well as their maintenance in the follow-up period, as observed in this study. Taken together, these results recommend the application of EME during the hamstring muscles stretching to enhance their flexibility [20,45].

The PNF group showed significant improvements in the AKE test after the intervention in the left limb, and increases were observed in both limbs in the SLR test. However, at the follow-up, both limbs evidenced higher flexibility in both the AKE and SLR tests. The mechanism of action of PNF has been attributed to the reciprocal inhibition of antagonist muscles, which allows greater stretching of the target muscle [47]. The latest systematic review [48] exploring the effects of PNF was only focused on its acute effects (i.e., the measurements were taken immediately after a single technique or session). Despite the temporal aspects differing from those evaluated here, it was concluded that a single PNF stretching session is sufficient to achieve an improvement in hamstring flexibility. Indeed, previous PNF programs developed with this aim have achieved success in the short, medium, and long term, which is consistent with the present findings [42,49,50,51]. The PNF technique could relax the connective tissue through both the stretching position and the hold–relax component, allowing a greater range of motion and thus improving muscle flexibility.

A secondary aim of this study was to explore how the quadricep muscles adapt to the stretching interventions that focus on their shortened antagonists. According to previous evidence [43], it was hypothesized that there was an increase in the quadricep flexibility following EME for stretching hamstrings. The present results confirmed this, also finding its maintenance in the follow-up, which is a novel report. Although the differences between groups were not statistically significant, the pattern observed for the increase in hamstring muscle flexibility was mostly replicated by the quadriceps: EME intervention showed generalized effectiveness, while partial (only distance buttock–heel) and unilateral (only left limb) effects were achieved after the PNF intervention, and both groups presented similarly increased quadricep flexibility in the follow-up. The phenomenon of reciprocal inhibition [37], the relaxation of the connective tissue, and the decrease in the mechanical stiffness of shared joints [52], as well as the peripheral and central neural plasticity [53] likely contributed to the parallel neuromuscular changes observed in the quadriceps. These findings are of particular interest and extend beyond hamstring injuries given the clinical relevance of both muscle group disorders in postural control [54], back pain [55], or sports biomechanics [56], among others. The sustained quadricep flexibility after EME suggests that the intervention not only provided immediate effects but also induced longer-term neuromuscular adaptations. EME could induce muscle relaxation and activate sensory fibers, allowing for deeper and more effective stretching. In addition, the low and medium-frequency electrical currents could modulate the viscoelastic properties of the muscles, improving their flexibility and maintaining these benefits in the long term.

Viscoelastic properties were also explored in both muscle groups. Regarding the hamstring muscles, despite no significant differences being found in the between-groups analysis, differentiated effects were observed at the intra-group level. The present results are consistent with the systematic review by Freitas et al. [57], showing that PNF alone does not produce changes in hamstring muscles stiffness, whereas this is the first study to assess changes in this structural parameter through a program based in EME for functional hamstring disorders. Specifically, a significant increase in stiffness was obtained after the EME intervention, which also persisted at the follow-up assessment. It should be noted that, although the recommendations for assessment through myotonometry were followed, several studies showed that stiffness responds in a non-homogeneous way at different hamstring muscle points [58,59]. A recent work [59] testing the biceps femoris in different zones after passive stretching demonstrated that the lower changes in stiffness were obtained in the midpoint site, where the measurements in the present study were taken. Therefore, the significant changes here observed in the EME group gain relevance. While one study highlighted that structural changes in viscoelastic properties leading to an increase in stiffness were washed shortly after the stretching [60], others found that this was achieved after a regular intervention of 4 to 6 weeks [59,61], as was the case in this study. However, according to a recent meta-analysis [18] on the effectiveness of stretching, little is known about the relevance of stiffness at the hamstring muscle bellies since the myotendinous unit is the most common site for injury. In the absence of normative data on this viscoelastic parameter, the present findings present new insights regarding the structural effects of applying EME during active stretching in the recovery of functional hamstring disorders. The addition of an interferential electrical current could further enhance hamstring muscle stiffness by promoting neuromuscular adaptation and increasing tissue resilience, which may contribute to injury prevention and improved functional performance.

Regarding quadricep muscle, significant differences in viscoelastic properties were only found in the right limb, with a decrease in stiffness in the EME group compared to the PNF and Control groups. Reciprocal inhibition, a spinal neurological mechanism whereby the activation and lengthening of one muscle group (hamstrings) induces the relaxation and stiffness reduction in the antagonist group (quadriceps) [62], is a likely explanation to the decrease achieved in quadricep stiffness while targeting hamstring muscles elongation. In contrast to the PNF intervention, in which only passive stretching and voluntary contractions are acting, the EME approach involves the added effects of the electrical current. The electrical stimulation may have triggered deeper neuromuscular circuits, leading to a stronger inhibitory effect on the quadriceps. Overall, the findings regarding stiffness support the fact that mechanical and neuromuscular stretching approaches can have different effects on the viscoelastic properties and recommend systematically assessing these structural changes both in the targeted and antagonist muscle groups [62,63].

This study has some limitations. The lack of stratification resulted in a non-homogeneous distribution of physical activity levels and lateral dominance in the study groups. Despite the absence of past strain-type injuries in the studied muscle groups as well as in related joints (lower back, hip, and knee) was guaranteed, any history of injuries in further structures may also impact findings on stiffness and flexibility, given the fascial continuum. In addition, the neurodynamic properties of the sciatic and femoral nerves were not evaluated and could impact hamstring and quadricep neuromuscular activity. However, several strengths are highlighted. We provide novel evidence of a stretching program based on EME improving hamstring flexibility in people with functional hamstring disorder, further maintaining these effects in the follow-up. Additional effects on hamstring stiffness and quadricep flexibility are informed. Given the feasibility and potential usefulness of EME, these findings need to be supported by clinical studies assessing their effectiveness in preventing the occurrence and recurrence of injuries.

## 5. Conclusions

The application of the EME technique can enhance the benefits of active stretching modalities such as PNF in young people with functional hamstring disorder when incorporated into a regular program. Improvements in muscle flexibility as well as viscoelastic changes were evidenced in the hamstring and quadricep muscles and persisted 4 weeks after the intervention.

## Figures and Tables

**Table 1:** Sociodemographic characteristics.

	EME Group (*n* = 21)	PNF Group (*n* = 22)	CT Group (*n* = 22)	*p* Value
Age (years)	23.45 ± 3.92	21.89 ± 3.58	23.71 ± 5.44	0.338 ^‡^
Gender (male)	16 (76.19%)	16 (72.72%)	13 (59.09%)	0.435 ^ƶ^
Height (m)	1.77 ± 0.09	1.77 ± 0.10	1.74 ± 0.09	0.647 *
Weight (kg)	71.95 ± 14.59	72.33 ± 15.58	68.74 ± 8.10	0.614 *
Body Mass Index (BMI) (kg/m^2^)	22.77 ± 2.91	22.22 ± 3.07	23.64 ± 2.33	0.257 *
Dominance (right)	16 (76.19%)	20 (90.90%)	22 (100%)	**0.040 ^ƶ^**
IPAQ				
Moderate	11 (52.38%)	5 (22.73%)	6 (27.27%)	**0.013 ^ƶ^**
High	10 (47.62%)	17 (77.27%)	16 (72.73%)	

Abbreviations: EME, Electrical Muscle Stretching; PNF, Proprioceptive Neuromuscular Facilitation; CT, Control; IPAQ, International Physical Activity Questionnaire. * Using one-way ANOVA; ^‡^ using Kruskal–Wallis test; ^ƶ^ using the chi-square test. Bold numbers indicate statistical significance.

**Table 2:** Hamstring flexibility.

Descriptive Data						Within-Groups Effect				Between-Groups Effect			
Variable			Pre-Test Mean ± SD Median [Interquartile Range]	Post-Test Mean ± SD Median [Interquartile Range]	Follow-Up Mean ± SD Median [Interquartile Range]	Post-Test vs. Pre-Test		Follow-Up vs. Pre-Test		Post-Test		Follow-Up	
						*p* Value	Effect Size	*p* Value	Effect Size	*p* Value	*p* Value (Only If Significant)–Effect Size	*p* Value	*p* Value (Only If Significant)–Effect Size
AKE test [°]	R	EME	46.85 ± 8.53	62.89 ± 9.47	59.74 ± 8.89	**<0.001** ^§^	**1.527**	**0.002** ^§^	**1.234**	0.305 ^‡^	EME-CT 0.622	0.429 ^‡^	EME-CT 0.394
			50 [30–60]	65 [50–80]	60 [50–80]								
		PNF	51.11 ± 8.14	57.50 ± 8.45	62.78 ± 10.74	0.067 ^§^	0.817	**<0.001** ^§^	**1.561**		PNF-CT 0.015		PNF-CT 0.687
			50 [40–70]	60 [40–70]	62.5 [40–80]								
		CT	53.10 ± 7.15	57.62 ± 7.35	56.66 ± 6.58	0.135 ^§^	0.552	0.244 ^§^	0.541		EME-PNF 0.601		EME-PNF 0.308
			55 [40–65]	55 [50–80]	56 [50–80]								
	L	EME	47.10 ± 8.71	63.16 ± 10.03	62.63 ± 6.32	**<0.001** ^§^	**1.326**	**<0.001** ^§^	**1.704**	**0.032** ^‡^	EME-CT **0.016** ^#^–**0.809**	0.456 ^‡^	EME-CT 0.785
			50 [30–60]	65 [40–80]	60 [50–75]								
		PNF	50.56 ± 7.05	59.44 ± 7.83	63.89 ± 8.67	**0.003** ^§^	**1.083**	**<0.001** ^§^	**1.444**		PNF-CT 0.067 ^#^–0.481		PNF-CT 0.835
			50 [40–70]	60 [40–70]	62.5 [50–80]								
		CT	49.05 ± 7.68	55.24 ± 9.55	56.29 ± 9.52	0.651 ^§^	0.524	0.124 ^§^	0.76		EME-PNF 0.248 ^#^–0.413		EME-PNF 0.166
			50 [30–60]	50 [40–80]	55 [30–70]								
SLR test [°]	R	EME	61.32 ± 7.23	80.26 ± 6.97	80.26 ± 7.35	**<0.001** ^§^	**1.92**	**<0.001** ^§^	**2.09**	0.243 ^‡^	EME-CT 1.316	0.151 ^‡^	EME-CT 1.336
			60 [50–75]	80 [70–90]	80 [70–90]								
		PNF	61.94 ± 9.87	75.83 ± 9.89	76.67 ± 9.24	**0.008** ^§^	**1.123**	**0.001** ^§^	**1.185**		PNF-CT 0.661		PNF-CT 0.729
			60 [40–80]	77.5 [50–90]	80 [50–90]								
		CT	64.29 ± 6.57	69.52 ± 9.20	70.71 ± 6.94	0.180 ^§^	0.568	0.102 ^§^	0.925		EME-PNF 0.518		EME-PNF 0.430
			65 [45–70]	70 [50–85]	70 [55–85]								
	L	EME	57.90 ± 9.33	78.95 ± 7.37	78.95 ± 7.37	**<0.001** ^§^	**1.983**	**<0.001** ^§^	**2.658**	0.098 ^‡^	EME-CT 0.732	0.821 ^‡^	EME-CT 0.787
			60 [40–75]	80 [70–95]	80 [70–90]								
		PNF	61.94 ± 8.07	78.61 ± 9.82	77.22 ± 8.26	**<0.001** ^§^	**2.028**	**0.001** ^§^	**1.436**		PNF-CT 0.629		PNF-CT 0.568
			60 [50–85]	80 [60–90]	80 [60–90]								
		CT	63.57 ± 5.04	71.90 ± 11.45	71.90 ± 10.30	0.092 ^§^	0.728	0.080 ^§^	0.809		EME-PNF 0.039		EME-PNF 0.221
			65 [50–70]	70 [50–90]	70 [60–90]								

Abbreviations: EME, Electrical Muscle Stretching Group; PNF, Proprioceptive Neuromuscular Facilitation Group; CT, Control Group; SD, Standard Deviation; R, right; L, left; ^§^ Using Friedman test; ^‡^ using Kruskal–Wallis test; ^#^ using Mann–Whitney U test. Bold numbers indicate statistical significance.

**Table 3:** Quadricep flexibility.

Descriptive Data						Within-Groups Effect				Between-Groups Effect			
Variable			Pre-Test Mean ± SD Median [Interquartile Range]	Post-Test Mean ± SD Median [Interquartile Range]	Follow-Up Mean ± SD Median [Interquartile Range]	Post-Test vs. Pre-Test		Follow-Up vs. Pre-Test		Post-Test		Follow-Up	
						*p* Value	Effect Size	*p* Value	Effect Size	*p* Value	*p* Value (Only If Significant) Effect Size	*p* Value	*p* Value (Only If Significant) Effect Size
Maximum flexion of the knee [°]	R	EME	139.21 ± 13.87	143.68 ± 9.40	143.16 ± 10.17	0.069 ^§^	0.397	0.186 ^§^	0.468	0.302 ^‡^	EME-CT 0.375	0.777 ^‡^	EME-CT 0.044
			140 [105–165]	145 [125–160]	140 [125–160]								
		PNF	141.67 ± 9.39	142.11 ± 7.95	142.78 ± 6.69	0.803 ^§^	0.057	0.453 ^§^	0.164		PNF-CT 0.210		PNF-CT 0.167
			142.5 [125–160]	140 [130–160]	142.5 [130–155]								
		CT	145.00 ± 9.22	140.48 ± 7.57	141.67 ± 6.58	0.076 ^§^	0.449	0.054 ^§^	0.427		EME-PNF 0.180		EME-PNF 0.174
			150 [120–160]	140 [130–160]	130 [130–155]								
	L	EME	135.79 ± 11.93	140.53 ± 10.12	140.53 ± 9.70	**0.045 ^§^**	**0.774**	0.086 ^§^	0.382	0.327 ^‡^	EME-CT 0.382	0.673 ^‡^	EME-CT 0.227
			135 [115–155]	140 [120–160]	140 [125–160]								
		PNF	139.72 ± 11.04	139.72 ± 9.15	140.28 ± 7.37	0.617 ^§^	0.026	0.617 ^§^	0.073		PNF-CT 0.309		PNF-CT 0.231
			140 [115–160]	140 [125–160]	140 [125–155]								
		CT	140.48 ± 9.86	137.14 ± 7.35	138.57 ± 7.44	0.076 ^§^	0.427	0.316 ^§^	0.297		EME-PNF 0.084		EME-PNF 0.029
			140 [115–165]	135 [125–155]	140 [125–155]								
Distance buttock–heel [cm]	R	EME	13.59 ± 7.96	9.74 ± 6.94	9.68 ± 7.09	**<0.001 ^†^**	**0.779**	**<0.001 ^†^**	**0.958**	0.604 *	EME-CT 0.224	0.882 *	EME-CT 0.115
			11.5 [0–33]	9.5 [0–22]	11 [0–23]								
		PNF	11.40 ± 6.52	9.89 ± 5.81	9.42 ± 6.60	0.287 ^†^	0.470	**0.043 ^†^**	**0.666**		PNF-CT 0.222		PNF-CT 0.168
			11.5 [0–23]	10 [0–19.5]	10 [0–23]								
		CT	10.58 ± 4.67	11.14 ± 5.45	10.36 ± 4.39	0.188 ^†^	0.436	0.862 ^†^	0.275		EME-PNF 0.023		EME-PNF 0.038
			10 [0–19]	11 [0–21.5]	11 [0–18]								
	L	EME	15.08 ± 7.71	12.03 ± 7.29	11.13 ± 7.02	**<0.001 ^†^**	**0.831**	**<0.001 ^†^**	**0.982**	0.555 *	EME-CT 0.113	0.817 *	EME-CT 0.074
			14 [0–29]	10 [0–24]	11 [0–26]								
		PNF	12.22 ± 7.57	11.39 ± 6.91	10.28 ± 7.05	0.999 ^†^	0.245	0.089 ^†^	0.613		PNF-CT 0.228		PNF-CT 0.205
			13.75 [0–28]	11.5 [0–25]	11 [0–26]								
		CT	11.79 ± 5.70	12.71 ± 5.69	11.60 ± 5.41]	0.922 ^†^	0.226	0.999 ^†^	0.051		EME-PNF 0.090		EME-PNF 0.121
			12 [0–24]	13 [0–23]	13 [0–21]								

Abbreviations: EME, Electrical Muscle Stretching Group; PNF, Proprioceptive Neuromuscular Facilitation Group; CT, Control Group; SD, Standard Deviation; R, right; L, left. ^†^ Using mixed-design ANOVA; ^§^ using Friedman test; * using one-way ANOVA; ^‡^ using Kruskal–Wallis test. Bold numbers indicate statistical significance.

**Table 4:** Hamstring muscle stiffness.

Descriptive Data						Within-Groups Effect				Between-Groups Effect			
Variable			Pre-Test Mean ± SD Median [Interquartile Range]	Post-Test Mean ± SD Median [Interquartile Range]	Follow-Up Mean ± SD Median [Interquartile Range]	Post-Test vs. Pre-Test		Follow-Up vs. Pre-Test		Post-Test		Follow-Up	
						*p* Value	Effect Size	*p* Value	Effect Size	*p* Value	*p* Value (Only If Significant) Effect Size	*p* Value	*p* Value (Only If Significant) Effect Size
Oscillation frequency [Hz]	R	EME	17.48 ± 1.59	18.63 ± 1.58	18.18 ± 1.48	**0.001 ^†^**	**0.728**	0.084 ^†^	0.285	0.172 *	EME-CT 0.597	0.163 *	EME-CT 0.401
			17.4 [14.6–21.1]	18.7 [16.2–22.5]	18.5 [15.4–20.9]								
		PNF	17.02 ± 1.90	17.39 ± 2.04	17.12 ± 2.27	0.483 ^†^	0.181	0.999 ^†^	0.119		PNF-CT 0.047		PNF-CT 0.122
			16.9 [13.4–21.5]	17.5 [13.5–20.8]	17.2 [12.6–20.4]								
		CT	16.93 ± 2.14	17.49 ± 2.19	17.40 ± 2.32	0.095 ^†^	0.256	0.290 ^†^	0.203		EME-PNF 0.680		EME-PNF 0.553
			17.1 [13.0–20.9]	17.9 [13.1–20.9]	17.5 [13.4–21.9]								
	L	EME	17.34 ± 1.40	18.19 ± 1.54	17.17 ± 1.40	**0.005 ^†^**	**0.552**	0.999 ^†^	0.121	0.177 *	EME-CT 0.916	0.346 *	EME-CT 0.369
			17.6 [15.3–20.1]	17.3 [15.2–20.3]	17.2 [14.4–19.9]								
		PNF	16.64 ± 2.16	16.86 ± 1.65	16.52 ± 2.20	0.999 ^†^	0.133	0.999 ^†^	0.055		PNF-CT 0.160		PNF-CT 0.009
			16.6 [12.4–20.6]	16.9 [13.1–19.9]	16.6 [12.1–19.9]								
		CT	16.96 ± 2.07	16.57 ± 1.97	16.50 ± 2.15	0.560 ^†^	0.198	0.231 ^†^	0.214		EME-PNF 0.833		EME-PNF 0.353
			16.5 [12.8–21.2]	16.6 [12.9–20.2]	16.7 [12.4–20.5]								
Decrement [arbitrary unit]	R	EME	1.29 ± 0.14	1.28 ± 0.17	1.29 ± 0.16	0.702 ^†^	0.059	0.999 ^†^	0.000	0.159 *	EME-CT 0.166	0.473 *	EME-CT 0.352
			1.26 [1.1–1.6]	1.29 [1.0–1.6]	1.21 [1.1–1.5]								
		PNF	1.24 ± 0.17	1.23 ± 0.18	1.27 ± 0.15	0.921 ^†^	0.056	0.300 ^†^	0.200		PNF-CT 0.108		PNF-CT 0.241
			1.29 [1.0–1.6]	1.21 [1.0–1.7]	1.20 [1.1–1.5]								
		CT	1.24 ± 0.18	1.25 ± 0.19	1.23 ± 0.18	0.999 ^†^	0.053	0.999 ^†^	0.056		EME-PNF 0.286		EME-PNF 0.129
			1.25 [0.9–1.6]	1.28 [0.8–1.6]	1.20 [0.9–1.7]								
	L	EME	1.28 ± 0.17	1.30 ± 0.17	1.30 ± 0.16	0.900 ^†^	0.118	0.882 ^†^	0.125	0.428 *	EME-CT 0.431	0.514 *	EME-CT 0.399
			1.29 [1.0–1.6]	1.24 [1.1–1.7]	1.24 [1.0–1.6]								
		PNF	1.23 ± 0.18	1.24 ± 0.17	1.27 ± 0.19	0.999 ^†^	0.056	0.594 ^†^	0.211		PNF-CT 0.108		PNF-CT 0.211
			1.21 [1.0–1.7]	1.24 [1.0–1.6]	1.29 [0.9–1.7]								
		CT	1.25 ± 0.19	1.22 ± 0.20	1.23 ± 0.19	0.892 ^†^	0.150	0.900 ^†^	0.105		EME-PNF 0.353		EME-PNF 0.171
			1.28 [0.8–1.6]	1.21 [1.0–1.7]	1.19 [1.0–1.7]								
Stiffness [N/m]	R	EME	328.88 ± 36.57	354.24 ± 37.10	342.76 ± 43.46	**0.002 ^†^**	**0.684**	0.207 ^†^	0.319	0.062 *	EME-CT 0.811	0.102 *	EME-CT 0.628
			333 [261–399]	349 [292–449]	341 [264–434]								
		PNF	314.56 ± 54.05	320.74 ± 53.11	317.95 ± 54.55	0.899 ^†^	0.116	0.914 ^†^	0.062		PNF-CT 0.058		PNF-CT 0.110
			307 [229–452]	311 [227–440]	311 [198–422]								
		CT	314.57 ± 44.27	317.71 ± 51.74	311.95 ± 54.06	0.921 ^†^	0.061	0.834 ^†^	0.048		EME-PNF 0.731		EME-PNF 0.503
			314 [219–386]	332 [291–394]	330 [199–414]								
	L	EME	324.71 ± 34.33	345.53 ± 35.17	326.29 ± 41.10	**0.005 ^†^**	**0.592**	0.999 ^†^	0.038	0.102 *	EME-CT 1.169	0.153 *	EME-CT 0.629
			320 [278–396]	341 [263–404]	327 [252–445]								
		PNF	307.32 ± 52.62	312.42 ± 41.62	307.42 ± 53.76	0.899 ^†^	0.123	0.999 ^†^	0.002		PNF-CT 0.359		PNF-CT 0.186
			300 [195–401]	314 [214–410]	316 [191–414]								
		CT	305.10 ± 48.03	296.29 ± 48.10	297.87 ± 48.86	0.587 ^†^	0.183	0.900 ^†^	0.148		EME-PNF 0.859		EME-PNF 0.394
			318 [213–420]	296 [199–376]	305 [187–371]								

Abbreviations: EME, Electrical Muscle Stretching Group; PNF, Proprioceptive Neuromuscular Facilitation Group; CT, Control Group; SD, Standard Deviation; R, right; L, left. ^†^ Using mixed-design ANOVA; * using one-way ANOVA; Statistical significance is indicated in bold.

**Table 5:** Quadriceps Muscle Stiffness.

Descriptive Data						Within-Groups Effect				Between-Groups Effect			
Variable			Pre-Test Mean ± SD Median [Interquartile Range]	Post-Test Mean ± SD Median [Interquartile Range]	Follow-Up Mean ± SD Median [Interquartile Range]	Post-Test vs. Pre-Test		Follow-Up vs. Pre-Test		Post-Test		Follow-Up	
						*p* Value	Effect Size	*p* Value	Effect Size	*p* Value	*p* Value (Only If Significant)–Effect Size	*p* Value	*p* Value (Only If Significant)–Effect Size
Oscillation frequency [Hz]	R	EME	15.68 ± 0.89	15.53 ± 1.12	15.28 ± 1.48	0.999 ^†^	0.134	0.268 ^†^	0.27	0.135 *	EME-CT 0.704	0.205 *	EME-CT 0.409
			15.8 [14.0–17.3]	15.6 [13.5–17.5]	15.4 [12.9–17.5]								
		PNF	14.75 ± 1.60	14.60 ± 1.55	14.64 ± 1.71	0.999 ^†^	0.097	0.999 ^†^	0.064		PNF-CT 0.007		PNF-CT 0.018
			14.4 [12.8–19.4]	14.3 [12.4–18.4]	17.4 [11.9–18.2]								
		CT	14.86 ± 1.61	14.59 ± 1.52	14.63 ± 1.69	0.582 ^†^	0.178	0.810 ^†^	0.136		EME-PNF 0.688		EME-PNF 0.425
			15.1 [11.8–17.4]	14.6 [12.3–17.3]	15.6 [11.9–17.3]								
	L	EME	15.10 ± 0.94	14.85 ± 1.22	14.59 ± 1.03	0.472 ^†^	0.205	0.059 ^†^	0.495	0.302 *	EME-CT 0.492	0.421 *	EME-CT 0.363
			15.3 [13.3–16.3]	14.0 [13.5–17.1]	14.4 [12.8–16.6]								
		PNF	14.65 ± 1.24	14.27 ± 1.44	14.33 ± 1.39	0.322 ^†^	0.264	0.229 ^†^	0.23		PNF-CT 0.008		PNF-CT 0.135
			14.6 [12.7–17.1]	13.9 [12.3–17.3]	14.3 [12.3–17.3]								
		CT	14.45 ± 1.63	14.26 ± 1.18	14.14 ± 1.42	0.999 ^†^	0.161	0.232 ^†^	0.218		EME-PNF 0.485		EME-PNF 0.218
			14.3 [11.5–17.1]	14.2 [12.2–16.8]	14.4 [12.0–16.9]								
Decrement [arbitrary unit]	R	EME	1.33 ± 0.19	1.43 ± 0.20	1.35 ± 0.16	0.095 †	0.5	0.999 ^†^	0.125	0.016 *	EME-CT 0.005 *–0.923	0.424 *	EME-CT 0 0.399
			1.32 [1.1–1.7]	1.45 [1.0–1.7]	1.36 [1.0–1.6]								
		PNF	1.30 ± 0.17	1.27 ± 0.20	1.32 ± 0.19	0.999 ^†^	0.15	0.999 ^†^	0.105		PNF-CT 0.513 *–0.103		PNF-CT 0.211
			1.24 [1.1–1.6]	1.21 [0.8–1.6]	1.28 [1.0–1.6]								
		CT	1.28 ± 0.23	1.25 ± 0.19	1.28 ± 0.22	0.939 ^†^	0.158	0.999 ^†^	0.136		EME-PNF 0.038 *–0.800		EME-PNF 0.171
			1.30 [0.7–1.6]	1.26 [1.0–1.6]	1.27 [0.8–1.7]								
	L	EME	1.38 ± 0.14	1.45 ± 0.17	1.43 ± 0.19	0.069 ^†^	0.412	0.388 ^†^	0.263	0.088 *	EME-CT 0.433	0.488 *	EME-CT 0.370
			1.35 [1.2–1.8]	1.48 [1.0–1.7]	1.39 [1.1–1.9]								
		PNF	1.34 ± 0.19	1.34 ± 0.22	1.39 ± 0.24	0.999 ^†^	0	0.538 ^†^	0.208		PNF-CT 0.087		PNF-CT 0.167
			1.33 [1.0–1.7]	1.37 [0.8–1.8]	1.42 [0.9–1.9]								
		CT	1.36 ± 0.28	1.36 ± 0.24	1.35 ± 0.24	0.999 ^†^	0	0.999 ^†^	0.042		EME-PNF 0.560		EME-PNF 0.185
			1.34 [0.8–2.0]	1.37 [1.0–1.9]	1.27 [1.0–2.1]								
Stiffness [N/m]	R	EME	280.94 ± 21.68	279.77 ± 30.34	282.76 ± 31.25	0.999 ^§^	0.039	0.899 ^§^	0.058	**0.011 ^‡^**	EME-CT **0.008** ^#^–**0.742**	**0.032 ^‡^**	EME-CT **0.009** ^#^**–0.638**
			277 [248–334]	280 [239–340]	282 [234–334]								
		PNF	267.90 ± 38.29	261.21 ± 35.40	263.32 ± 37.45	0.999 ^§^	0.189	0.999 ^§^	0.122		PNF-CT 0.782 ^#^–0.135		PNF-CT 0.999 ^#^**–**0.034
			265 [224–394]	263 [205–356]	259 [204–341]								
		CT	259.81 ± 36.27	256.67 ± 31.91	262.10 ± 33.43	0.999 ^§^	0.098	0.999 ^§^	0.069		EME-PNF **0.010** ^#^–**0.563**		EME-PNF 0.053 ^#^**–**0.564
			266 [175–311]	259 [184–313]	330 [189–322]								
	L	EME	279.82 ± 25.42	276.53 ± 31.91	268.12 ± 29.25	0.999 ^†^	0.103	0.240 ^†^	0.4	0.040 *	EME-CT 0.049 *–0.636	0.173 *	EME-CT 0.556
			288 [226–317]	274 [222–333]	265 [214–316]								
		PNF	263.26 ± 22.96	253.10 ± 32.93	256.16 ± 33.12	0.177 ^†^	0.309	0.318 ^†^	0.214		PNF-CT 0.575 *–0.169		PNF-CT 0.140
			264 [228–305]	252 [207–313]	265 [201–321]								
		CT	253.95 ± 32.85	258.10 ± 25.73	251.76 ± 29.63	0.999 ^†^	0.226	0.999 ^†^	0.074		EME-PNF 0.024 *–0.723		EME-PNF 0.383
			253 [191–308]	258 [206–330]	255 [203–314]								

Abbreviations: EME, Electrical Muscle Stretching: PNF, Proprioceptive Neuromuscular Facilitation; CT, Control; SD, Standard Deviation; R, right; L, left. ^†^ using mixed-design ANOVA; ^§^ using Friedman test; * using one-way ANOVA; ^‡^ using Kruskal-Wallis test; ^#^ using Mann–Whitney U test. Bold numbers indicate statistical significance.

## Data Availability

The data can be provided upon a reasonable request to the corresponding author.
